# Myocardial oxidative damage is induced by cardiac Fas-dependent and mitochondria-dependent apoptotic pathways in human cocaine-related overdose

**DOI:** 10.1038/srep44262

**Published:** 2017-03-10

**Authors:** Emanuela Turillazzi, Daniela Cerretani, Santina Cantatore, Anna Ida Fiaschi, Paola Frati, Lucia Micheli, Margherita Neri, Luigi Cipolloni, Marco Di Paolo, Enrica Pinchi, Irene Riezzo, Alessandro Santurro, Annamaria Vullo, Vittorio Fineschi

**Affiliations:** 1 Institute of Legal Medicine, Department of Clinical and Experimental Medicine, University of Foggia – Viale Pinto 1, 71121 Foggia, Italy; 2 Department of Medical and Surgical Sciences and Neurosciences, University of Siena – Viale Mario Bracci 16, 53100 Siena, Italy; 3 Department of Anatomical, Histological, Forensic and Orthopaedic Sciences, S apienza University of Rome – Viale Regina Elena 336, 00185 Rome, Italy; 4 Neuromed, Istituto Mediterraneo Neurologico (IRCCS) – Via Atinense 18, Pozzilli, 86077 Isernia, Italy; 5 Department of Morphology, Experimental Medicine and Surgery, University of Ferrara, Via Fossato di Mortara 70, 44121 Ferrara, Italy; 6 Institute of Legal Medicine, Department of Clinical and Experimental Medicine, University of Pisa – Via Roma 55, 56126 Pisa, Italy

## Abstract

The aim of this study is to analyse cardiac specimens from human cocaine-related overdose, to verify the hypothesis that cardiac toxicity by acute exposure to high dosage of cocaine could be mediated by unbalanced myocardial oxidative stress, and to evaluate the apoptotic response. To address these issues, biochemical and immunohistological markers of oxidative/nitrosative stress were evaluated. We found that i-NOS, NOX2 and nitrotyrosine expression were significantly higher in the hearts of subjects who had died from high doses of cocaine, compared to the control group. Increase of these markers was associated with a dramatic increase in 8-OHdG, another marker of oxidative stress. A high number of TUNEL-positive apoptotic myocells was observed in the study group compared to the control group. The immunoexpression of TNF-α was significantly higher in the cocaine group compared to the control group. Furthermore, we detected a significantly stronger immunoresponse to anti-SMAC/DIABLO in our study group compared to control cases. Both cardiac Fas-dependent and mitochondria-dependent apoptotic pathways appeared to be activated to a greater extent in the cocaine group than in the control group. Our results highlight the central role of oxidative stress in cocaine toxicity. High levels of NOS can promote the oxidation process and lead to apoptosis.

Cocaine, also known as benzoyl methyl ecgonine, is an alkaloid extracted from erythroxylon coca leaves which is widely abused worldwide^1^. Acute and chronic cocaine use is responsible for a variety of systemic complications that have been reported in nearly every organ and system: the brain, heart, lungs, kidneys, gastrointestinal tract, musculature, and other organs may be involved^2^.

In particular, cocaine can cause a kaleidoscope of cardiac pathologies^3^ and, as its abuse has become widespread, the number of cocaine-related cardiovascular adverse events has dramatically increased^4^,^5^,^6^. The plethora of cocaine-related cardiovascular complications, both acute and chronic, include acute myocardial ischemia and infarction, arrhythmias, sudden death, myocarditis, cardiomyopathy, hypertension, aortic ruptures, and endocarditis^7^,^8^,^9^,^10^,^11^.

The cardiac effects of cocaine are complex, and our understanding of the mechanisms of cocaine cardiotoxicity is far from complete. Cocaine cardiotoxicity has long been thought to be mediated indirectly through its sympathomimetic effect, i.e., by inhibiting the presynaptic reuptake and increasing the levels of neuronal catecholamines (dopamine and norepinephrine) with a resulting increase in their concentration in the synaptic cleft and enhanced post-synaptic transmission, as well as enhanced central sympathetic outflow^12^. The mechanisms of cocaine cardiotoxicity further include blockage of sodium and potassium channels, disruption of excitation-contraction coupling, and altered calcium flux across myocyte cell membrane^13^.

In recent years, an important area of study has addressed the sources and effect of reactive oxygen species (ROS) and reactive nitrogen species (RNS) in heart diseases, both of which are considered major biologically relevant redox active molecules^14^. Accumulating evidence suggests that cocaine administration is associated with severe nitrosative/oxidative stress and mitochondrial dysfunction of the heart, and experimental studies have reported altered oxidative balance in the myocardium of chronic cocaine-treated animals^15^,^16^,^17^,^18^,^19^,^20^,^21^,^22^,^23^.

The adrenergic over-stimulation induced by cocaine is correlated to its ability to increase oxidative stress and several mechanisms have been proposed. Previous studies have shown that oxidation of catecholamines results in the formation of highly toxic substances such as aminochromes (e.g. adrenochrome). Adrenochrome is a likely candidate for such a process of redox cycling, leading to the formation of ROS. Acting on different types of heart membranes, ROS cause depletion of cellular antioxidants (e.g. ascorbic acid, AA; glutathione, GSH), intracellular Ca^2+^ overload, lipid peroxidation and myocardial cell damage^24^.

Recently it has been hypothesized that oxidative stress could play a significant role in the pathogenesis of cardiotoxicity in chronic cocaine abusers^23^,^25^,^26^. It is noteworthy that ROS, generated during oxidative stress, are responsible for the pathophysiology of various cardiovascular disorders including atherosclerosis, cardiac hypertrophy, cardiomyopathy, heart failure, ventricular remodelling, ischemia/reperfusion injury and myocardial infarction^27^. Direct cocaine activation of NADPH (nicotinamide adenine dinucleotide phosphate) oxidases (NOX), secondary activation of xantine oxidase, formation of oxidative metabolites, and adrenoceptor hyperstimulation with auto-oxidation of cathecolamines are the hypothesized sources of cocaine-induced ROS in cardiomyocytes^26^. Even though various studies report the deleterious effects of chronic cocaine assumption on the oxidative balance of the heart, there is a lack of information in the literature about the effects of acute high dosage of cocaine on cardiac oxidative homeostasis.

In the light of these previous findings, this study reports an evaluation of myocardial oxidative damage, analysing cardiac specimens from subjects who have died suddenly from acute cocaine intoxication. This is in order to verify the hypothesis that cardiac toxicity caused in humans by acute exposure to high doses of cocaine could be mediated, at least in part, by unbalanced myocardial oxidative stress. To this end, we selected cases of young people who had died from a high-level dose of cocaine, for which the term overdose is correctly used. To address these issues, biochemical and immunohistological markers of oxidative/nitrosative stress were evaluated.

## Results

### Toxicological analysis

All the cases are body packers, i.e. international smugglers who insert or ingest into body orifices small packages of drugs for the purposes of the transport and subsequent retrieval of the drug in a foreign country. All the subjects died from cocaine intoxication and the cocaine blood level at the time of death varied as indicated in Table 1.

In four cases, cocaine was associated only with ethyl alcohol. The levels of alcohol found in the blood ranged from trace to 0.8 g/l. In no case was a concentration of other drugs found in the blood or urine at the time of death considered to be responsible for, or have contributed to death.

### Biochemical analysis

To determine the degree of oxidative stress in study-group cardiac specimens, the levels of lipid peroxidation and ascorbic acid, and GSH/GSSG ratio were measured and the results are shown in Table 2.

**Figure 1:**
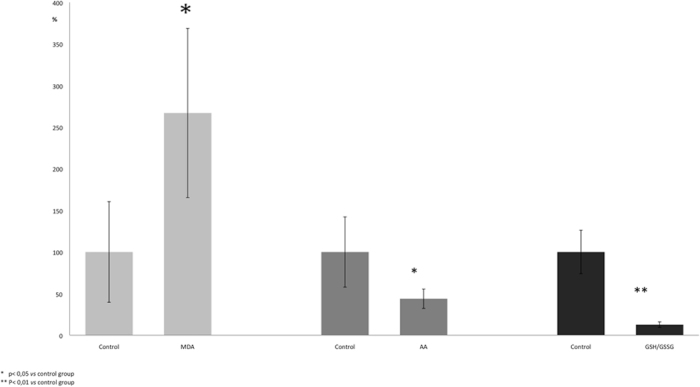
The level of MDA was significantly elevated (p < 0.05) in study group cardiac specimens compared to the control group. Cardiac cytosolic levels of AA were significantly lower (p < 0.05) and the GSH/GSSG ratio was dramatically lower (p < 0.01) in the study group cases compared to controls. Results were expressed as mean ± SD. The comparison between groups was conducted using the comparison Student’s *t* test. A value of *p* < 0.05 was considered statistically significant. *p < 0.05; **p < 0.01 study group vs control group.

The level of MDA, an indicator of lipid peroxidation, was significantly elevated (p < 0.05) in study group cardiac specimens compared to the control group. Cardiac cytosolic levels of AA were significantly lower (p < 0.05) in the study group compared to controls. Furthermore, the GSH/GSSG ratio was dramatically lower (p < 0.01) in study group cases compared to controls (Fig. 1).

The analysis of these parameters highlights the presence of high oxidative stress in examined cardiac samples.

### Histology and immunohistochemistry

The histological and immunohistochemical characteristics of the study group compared to the control group are shown in Table 3.

**Figure 2:**
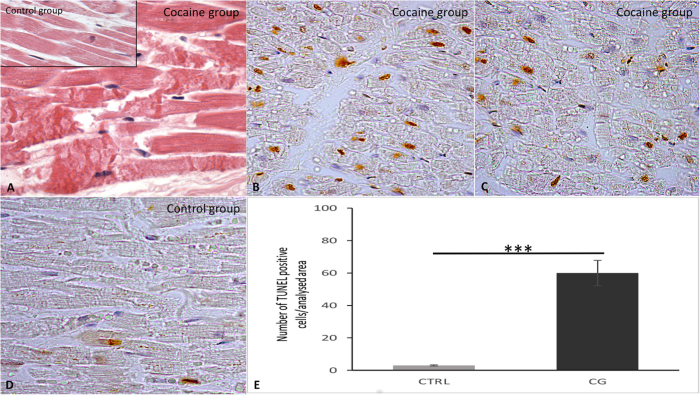
(**A**) Hypereosinophilic, hypercontracted myocardial cells with rhexis of the myofibrillar apparatus into cross-fibre, anomalous, and irregular bands (insert: normal aspect of myocytes in control group specimens) (haematoxylin and eosin, original magnification 100x). (**B,C**) Increase of apoptotic cells was measured by TUNEL assay: values of myocyte cell apoptosis in the cocaine group were significantly higher than control cases. The percentage of apoptotic myocyte nuclei was determined. Apoptosis was measured by the determination of the fraction of myocyte nuclei labelled by TUNEL: 60 ± 13% and 3 ± 1% apoptotic cells were observed in cocaine group and control group (**D**) (original magnification for B-C-D 63x), respectively. (**E**) Quantification of apoptosis positive-stained nuclei between groups: the percentage of apoptotic myocyte nuclei was significantly elevated (p < 0.001) in study group specimens compared to the control group. Values are presented as mean ±SD (standard deviation). The unpaired two-way Student’s t-test was used to compare the results obtained for cocaine group with the control group. p < 0.05 was accepted as indicative of significant difference among groups.

The main change noted during histological observation was the presence of hypereosinophilic, hypercontracted myocardial cells with rhexis of the myofibrillar apparatus into cross-fibre, anomalous, and irregular bands (contraction band necrosis, CBN). This necrosis was, in general, plurifocal and formed by foci found in cardiac specimens of the study group (Fig. 2A). Semi- quantitative analysis showed that the amount of CBN foci was higher in the study group than in the control group.

Apoptosis was measured by TUNEL assay and values of myocyte cell apoptosis in the study group were significantly higher than control cases. The apoptotic nuclei of myocytes appeared isolated or irregularly distributed over the entire cardiac section from the subepicardium to subendocardium. The percentage of apoptotic myocyte nuclei was determined. The immunohistochemical study revealed an intensive positive result to TUNEL assay (Fig. 2B,C): 60 ± 13% and 3 ± 1% apoptotic cells were observed in cocaine group and control group, respectively.

**Figure 3:**
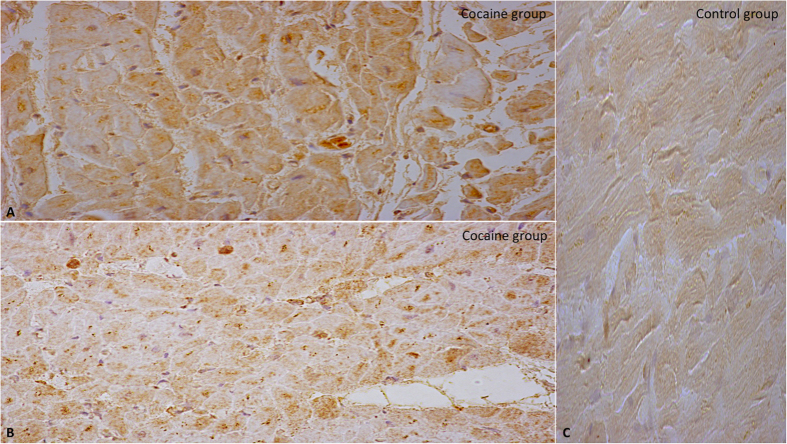
Intense and massive positive (brown in A and in B) myocyte immunoreaction of NOX2 (**A,B**) in the cocaine group, with representative images of NOX2 immunostaining demonstrated by brown reaction in the cardiac cells compared by the negativity in the control group (**C**) (original magnification for A-B-C 40x).

**Figure 4:**
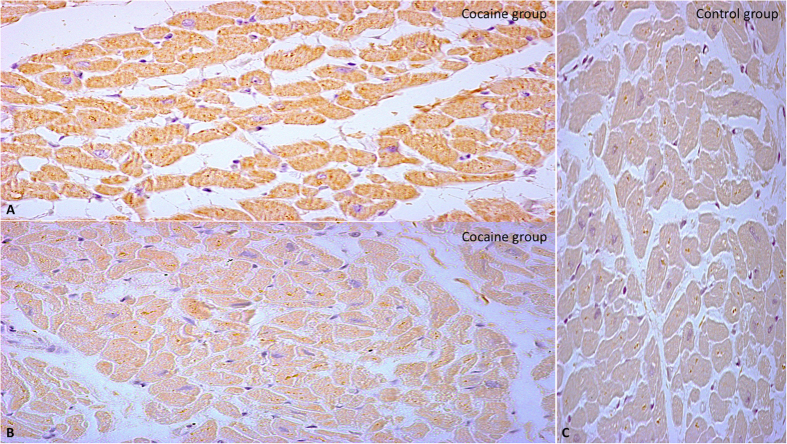
(**A,B**) Immunohistochemistry analysis of i-NOS showing positive staining (yellow reaction) in cocaine group heart tissue and compared with the control group (original magnification for A-B-C 40x) (**C**).

Semi-quantitative evaluation (grade 0–4) revealed increased myocyte immunoreaction of NOX2 (Fig. 3).

i-NOS expression was significantly increased in the hearts of cocaine group with respect to control group subjects whose death was from other causes (Fig. 4).

**Figure 5:**
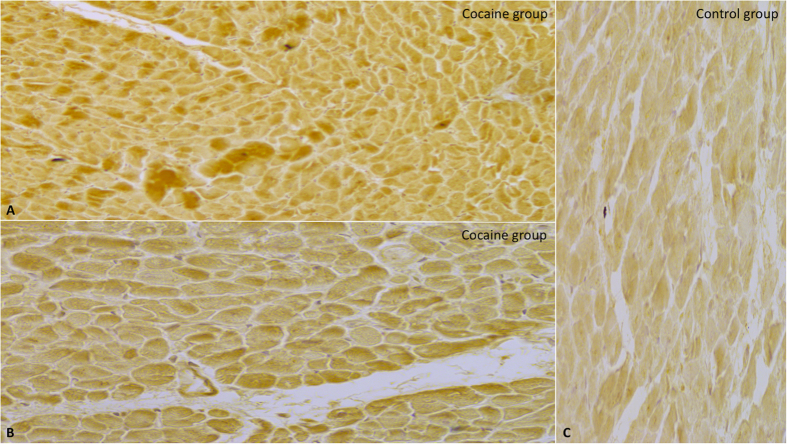
Intense nitrotyrosine reaction (darker reaction in cardiac cell) in cocaine group study (**A,B**), compared with control group (original magnification for A-B-C 40x) (**C**).

Elevation of the nitrotyrosine markers was documented in the cocaine overdose group with respect to control group (Fig. 5).

**Figure 6:**
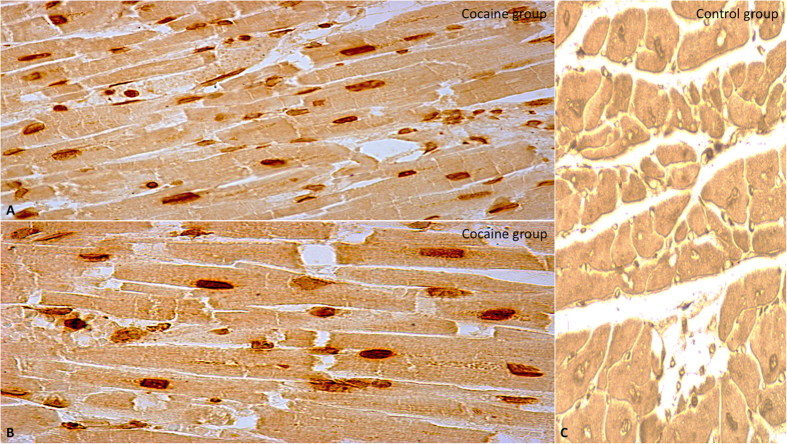
8-OHdG positive staining in about 70% of cardiomyocytes nuclei (nuclear brown reaction) from study group specimens (**A,B**), while they were detected in less than 5% in control group cardiac specimens (**C**) (original magnification for A-B-C 80x). Values are presented as mean ±SD (standard deviation). The unpaired two-way Student’s t-test was used to compare the results obtained for cocaine group with the control group. p < 0.05 was accepted as indicative of significant difference among groups.

Positive 8-OHdG staining was found in 70% of cardiomyocytes nuclei from study group specimens while they were not detected in control group cardiac specimens (Fig. 6).

**Figure 7:**
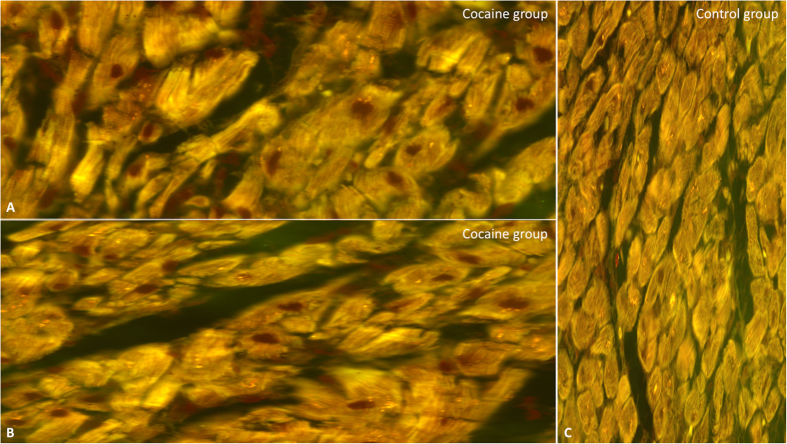
SMAC Diablo immunostaining in cardiomyocytes nuclei (nuclear brown reaction) from cocaine group specimens (**A,B**), while they were detected in less than 5% in control group cardiac specimens (**C**) (original magnification for A-B-C 100x).

**Figure 8:**
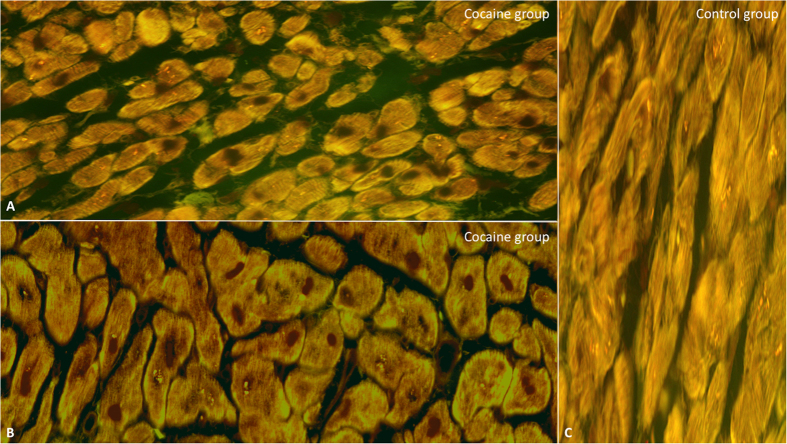
(**A,B**) Increase of Bcl-2 positive (nuclear brown reaction) immunostaining in the cells’nuclei of the cocaine group; positive cardiomyocytes nuclei were significantly higher than control case group (**C**) (original magnification for A-B-C 80x).

Strong TNF-α and SMAC/DIABLO positivity and weak Bcl-2 immunostaining were detected in cardiac specimens of the study group in respect to the control group (Figs 7 and 8).

## Discussion

In this study, we evaluated a possible increase in oxidative/nitrosative stress in the cardiac samples of subjects who had died following the assumption of high, lethal doses of cocaine. We found i-NOS, NOX2 and nitrotyrosine expression to have significantly increased in the hearts of these subjects with respect to control group subjects whose death was from other causes. The elevation of these markers was associated with a dramatic increase in another marker of oxidative stress, 8-OHdG, thus supporting the fact that an imbalance in oxidative/nitrosative stress is a key factor in cardiac acute cocaine-induced toxicity.

Our results are in line with previous basic and clinical studies that have highlighted the central role of oxidative/nitrosative stress in cocaine toxicity^15^,^16^,^17^,^18^,^19^,^20^,^21^,^22^,^23^.

**Figure 9:**
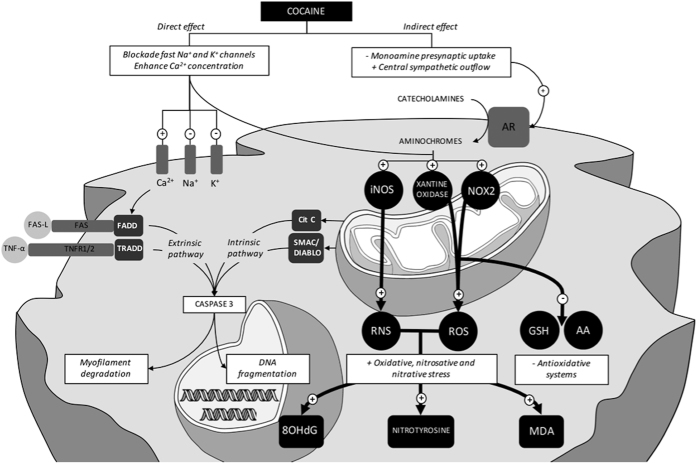
Mechanisms of the pathological effects of cocaine: apoptotic myocells process is due both to cardiac Fas-dependent and mitochondria-dependent apoptotic pathways. Evaluation of myocardial oxidative damage is the hypothesis that cardiac toxicity caused in humans by acute exposure to high dosage of cocaine could be mediated, at least in part, by unbalanced myocardial oxidative stress.

Most toxic effects of cocaine on molecular levels are mediated by oxidative stress or mitochondrial dysfunction caused by metabolization of noradrenalin or norcocaine^23^,^28^,^29^. Catecholamines are transformed into ‘aminochromes’, which may undergo redox cycling after entering the mitochondria. This leads to the production of a large quantity of ROS^29^ that can deplete cellular antioxidants such as AA and GSH. In cardiomyocytes and many mammalian cells, GSH, in a reduced form, is considered the major cytosolic redox buffer. When oxidative stress occurs, the GSH/GSSG ratio can rapidly decrease^30^. AA is a water-soluble electron transport antioxidant present in the cytosolic and in extracellular fluid and considerable attention has been given to its role in the cardiovascular event^31^. It is able to scavenge a wide variety of radicals, specifically superoxide anion radicals, peroxyl-derived radicals, and hydroxyl radicals. Previous studies have established that GSH is essential for the physiological function of AA because it is required for the reduction of dehydroascorbic acid and there is metabolic redundancy and overlap of the functions of these antioxidants. In fact, AA can spare GSH and thus protect against the effects of GSH deficiency. Although GSH and AA can perform functions in common, each one is likely to participate in reactions that the other cannot do efficiently^32^,^33^. The results of our study show a dramatic decrease in the GSH/GSSG ratio and in AA levels combined with an increase in MDA concentrations in cardiac samples of body-packers. This unequivocally shows that high oxidative stress occurred. In turn, calcium overload and oxidative stress promote mitochondrial permeability transition and cardiomocyte death via activation of both the apoptotic and necrotic pathways^28^. High levels of NOS can promote the oxidation process and lead to apoptosis^34^. The pivotal role of mitochondrial oxidative stress in cocaine-induced cardiotoxicity is also confirmed by a study by Vergeade *et al*. which demonstrates a decrease in cardiotoxicity by MitoQ^21^, a mitochondrial target antioxidant^28^ (Fig. 9).

Conclusively, emerging evidences converge towards the role of oxidative stress imbalance in chronic cocaine toxicity.

This is why it is important to investigate the alterations of nitric oxide (NO) and oxidative stratus in the heart after acute, massive cocaine assumption. NO synthesis is activated by one of the three isoforms of nitric oxide syntase (NOS) that are obligate homodimers catalyzing NADPH-dependent oxidation of L-arginine to NO and L-citrulline: NOS1 (neuronal or n-NOS), NOS2 (inducible or i-NOS), and NOS3 (endothelial or e-NOS)^35^.

i-NOS produces NO in response to a wide array of stimuli, mostly endotoxin and endogenous pro-inflammatory mediators. Moreover, cocaine enhances NOS levels in different organs and also in plasma^36^. Methylecgonidine (the principal pyrolysis product of crack cocaine base) has been shown to enhance NO production in cultured neonatal rat cardiomyocytes^37^. The spectrum of actions of NO is quite complex, and its interactions with oxygen or oxygen-related reactive intermediates (e.g. superoxide) yield numerous ROS and RNS. These account for most of the so-called indirect effects attributed to NO through oxidation, nitrosation, and nitrate reactions referred to as oxidative, nitrosative and nitrative stress, respectively^38^.

NO influences several aspects of cardiomyocytes functioning. The physiological production of NO in the heart maintains coronary vasodilator tone, and inhibits platelet and neutrophil adhesion, thus performing an active role in cardioprotection^39^. Excessive NO formation is thought to contribute to contractile dysfunction^40^,^41^. The role of i-NOS in cardiac function during the development of left ventricular hypertrophy in mice has been investigated, and recent data demonstrate that NO production via i-NOS plays an important role in modulating cardiac function after moderate aortic banding which mimics long-term hypertension in humans^42^.

Peroxynitrite (ONOO^−^), which derives from the reaction between NO and superoxide anions is a potent, citotoxic oxidant capable of inducing all three forms of stress mentioned above. Peroxynitrite-mediated nitrative stress induces severe damage to proteins, lipids, and DNA, resulting in cell damage or apoptosis and cytotoxicity. Nitrotyrosine (NT) formation has been used extensively as a marker of the generation of peroxynitrite^43^,^44^,^45^,^46^,^47^,^48^.

Interestingly, it has been recently shown that oxidative myocardial damage in human cocaine-related cardiomyopathy is mediated by increased NT production^26^.

Here we present our results showing significantly increased cardiac immunoreactions to NT and i-NOS after massive assumption of cocaine, suggesting an additional mechanism of acute cardiac injury from cocaine.

To obtain further insight into cocaine myocardial damage we explored the expression of NOX2 in cardiomyocytes. Other sources of ROS production are NADPH oxidases (NOX). These enzymes may be responsible for large amounts of superoxide and hydrogen peroxide production in several pathological conditions. The family of NOX proteins plays an integral role in the homeostatic functions of the cell, including gene expression, cell migration, proliferation, senescence and inflammation. The balance between NOX-derived reactive oxygen species production and their elimination by dismutase enzymes is a critical finely-tuned process. The role of NOX2 in human pathologies has been well-documented for specific diseases^49^, such as diabetes^50^,^51^,^52^,^53^,^54^. An increase in the expression of NOX2 in human cardiomyocytes in acute myocardial infarction has been detected^55^. Finally, chronic binge cocaine consumption has been demonstrated to significantly up-regulate the protein transcription of NOX2 and its component and strongly promote ROS production from NOX2 oxidase^20^.

Our results showed a significant increase in the immunoexpression of NOX2 in the study group in respect to control cases, thus confirming an imbalance of oxidative status.

Oxidation damage to DNA is the most detrimental, since replication of damaged DNA can lead to genetic mutations or apoptosis^56^,^57^. 8-hydroxy-2′-deoxyguanosine (8-OHdG) is a marker of oxidative DNA damage^58^,^59^,^60^,^61^. Recently the association between 8-OhdG and cardiovascular diseases has been reviewed^62^, and high levels of this marker in blood and urine have been associated with cardiovascular disease, such as atherosclerosis, heart failure, and ischemic/reperfusion injury following acute myocardial infarction^63^.

In this study, we assessed the level of DNA oxidative stress by detecting the expression of 8-OHdG. It was found that the positive rate of 8-OHdG expression in myocardial nuclei was higher in the study group than in the control group.

Finally, we explored myocyte apoptosis in our study group compared with control cases.

Apoptosis, a programmed, physiological cellular death, may contribute to many cardiac disorders. Studies have shown that oxidative stress may result in cardiomyopathy through activation of the apoptosis programme in myocardial cells^64^. Our group’s previous studies indicated that chronic cocaine exposure induces apoptosis in the heart^3^,^22^,^24^. Furthermore, in-utero cocaine exposure induces cardiac myocyte death in experimental animal models^65^,^66^,^67^. Both the intrinsic and extrinsic apoptotic pathways are involved. While previous studies suggest that cocaine-induced apoptosis in cardiomyocytes is mediated only by the mitochondria-dependent, intrinsic, pathway^6^,^9^,^68^, Liou *et al*.^69^ recently demonstrated that chronic cocaine exposure appeared to also activate the cardiac Fas- dependent, extrinsic apoptosis.

To investigate the two apoptotic pathways in our study group, TNF-α (an upstream component of cardiac Fas-dependent apoptotic signalling pathway), Bcl-2 and SMAC/Diablo (as markers of mitochondria-dependent apoptotic pathway) were examined.

The immunoexpression of TNF-α was significantly higher in the cocaine group compared to the control group, thus suggesting that also in acute cardiac toxicity from cocaine, the Fas-dependent pathway could be a key factor in triggering cardiac apoptosis.

Furthermore, we detected a significantly stronger immunoresponse to antibody anti-SMAC/DIABLO in our study group compared to control cases, thus suggesting the possibility that mitochondrial-induced apoptosis could be triggered in acute cardiotoxicity from cocaine.

SMAC/DIABLO is a mitochondria-derived pro-apoptotic molecule that appears to function by neutralizing the caspase-inhibitory properties of the IAP family of proteins, particularly XIAP^70^,^71^. Similarly to cytochrome *c*, SMAC/DIABLO is encoded by a nuclear gene and is subsequently imported into mitochondria. Mitochondrial SMAC/DIABLO release is a general feature of apoptosis, and Bcl-2 regulates this event. In cells stimulated to die in response to diverse pro-apoptotic agents, including death receptor ligation, cytotoxic drugs and DNA-damaging agents, SMAC/DIABLO accumulation within the cytosol was readily detected. This coincided with a concomitant loss of SMAC/DIABLO from mitochondria. It was demonstrated that Bcl-2 blocks apoptosis-associated release of SMAC/DIABLO from mitochondria, confirming the anti-apoptotic roles of Bcl-2^72^.

## Conclusions

Conclusively, our main findings can be summarized as follows: 1) a great number of TUNEL-positive apoptotic myocells was observed in the study group compared to the control group; 2) both cardiac Fas-dependent and mitochondria-dependent apoptotic pathways appeared to be more activated in the cocaine group compared to the control group; 3) myocardial oxidative damage, the evidence for which is based on increases in i-NOS, NOX2, NT, and 8-OHdG expression and an increase in the biochemical marker of lipid peroxidation (MDA) and in the depletion of antioxidant reserve as expressed by AA levels and GSH/GSSG ratio.

## Material and Methods

### Study population

The processing of the data reported in this paper is covered by the general authorization to process personal data for scientific research purposes granted by the Italian Data Protection Authority (1 March 2012 as published in Italy’s Official Journal no. 72 dated 26 March 2012) since the data do not entail any significant personalized impact on data subjects. Our study does not involve the application of experimental protocols; therefore it does not require approval by an institutional and/or licensing committee. In all cases, local prosecutors opened an investigation, ordering that an autopsy be performed to clarify the exact cause of death. From the autopsies performed at our departments between January 2001 and December 2014, nine cases (six males, three females, mean age 28.1 ± 10,9 years) of acute cocaine intoxication (body-packers and body-stuffers) were selected. These nine subjects comprise our study population (study group). Cases with toxicological findings suggestive of polysubstances abuse were excluded. Results were compared with cardiac samples from nine subjects matched for age and sex, who had died suddenly from traumatic causes (control group) in which toxicological analyses were negative.

### Toxicological analysis

Toxicological analyses by solid-liquid extraction and gas chromatography-mass spectrometry (GC-MS) were carried out to identify and quantify any substance present in biological fluid and organs.

### Biochemical analysis

#### Malondialdehyde (MDA) assessment

The extent of lipid peroxidation, a marker of oxidative stress, was estimated using MDA level calculation. Cardiac samples were homogenized in a 0.04 M K^+^ phosphate buffer (pH 7.4) containing 0.01% butyl hydroxytoluene (BHT) (1:5 w/v, 0 °C) to prevent the artificial oxidation of polyunsaturated free fatty acids during the assay. This homogenate was deproteinised with acetonitrile (1:1) and then centrifuged at 3000 g for 15 min. The supernatants were used for MDA-analysis after pre-column derivatization with 2.4-dinitrophenylhydrazine. The MDA-hydrazone was quantified by isocratic reversed-phase HPLC method with UV detection as described by Shara *et al*.^73^.

#### Reduced glutathione (GSH) and oxidized glutathione (GSSG) determination

Reduced glutathione (GSH) is considered to be one of the most important scavengers of ROS, and its ratio to oxidised glutathione (GSSG) may be used as a marker of oxidative stress. Cardiac tissue was homogenized in ethylenediaminetetraacetic acid (EDTA) K^+^ phosphate buffer, pH 7.4 (1:3, w/v) at 0 °C and 1 ml aliquots of the samples were added to an equal volume of 25% tricholoroacetic acid (TCA). After centrifugation at 2000 g for 15 min (0 °C), the supernatant was washed with diethyl ether. The levels of total GSH were measured using an enzyme-recycling assay based on the colorimetric reaction of GSH with DTNB in the presence of excess glutathione reductase and NADPH. TNB chromophore formation was followed spectrophotometrically at 405 nm. Total GSH was analyzed as described by Tietze^73^ and GSSG was determined according to Griffith’s method^74^.

#### Ascorbic Acid (AA) assay

Cardiac tissues were homogenized in EDTA-K^+^ phosphate buffer pH 7.4 (1:4 w/v) at 0 °C and analysed as described by Ross^75^. Samples (0.6 ml) were added to an equal volume of 10% (w/v) metaphosphoric acid and immediately centrifuged at 2000 × g at 0 °C for 10 min. Ascorbic acid was determined with a simple method by reversed-phase HPLC using an ion-pairing reagent with UV detection at 262 nm.

#### Histological and immunohistochemical study

In each case, tissue samples were obtained from the heart (seven standard specimens). Paraffin- embedded heart specimens were sectioned at 4 μm and stained with haematoxilin, eosin and Masson trichrome. In addition, an immunohistochemical study was performed with antibodies anti-NT (nitrotyrosine, Santa Cruz, CA, USA), anti-NOX2 (nicotinamide adenine dinucleotide phosphate (NADPH) oxidase 2, Santa Cruz, CA, USA), anti-i-NOS (inducible Nitric oxide synthase, Santa Cruz, CA, USA), anti-8-OHdG (8-Hydroxy-2′-deoxyguanosine, JaICA, Japan), anti-TNF-α (tumour necrosis factor-α, Santa Cruz, CA, USA), anti-BCL2 (B-cell lymphoma 2, Chemicon, Millipore, Billerica, MA, USA), anti-SMAC/DIABLO (second mithocondria-derived activator of caspase/direct IAP binding protein with low PI, Chemicon, Millipore, Billerica, MA, USA), and apoptosis with TUNEL assay (Apotag Plus Peroxidase *In Situ* Apoptosis Detection Kit, Chemicon, Millipore, Billerica, MA, USA).

Briefly, we used 4 μm-thick paraffin sections mounted on slides covered with 3, aminopropyltriethoxysilane (Fluka, Buchs, Switzerland). Pre-treatment was necessary to facilitate antigen retrieval and to increase membrane permeability to antibodies anti-TNF- α, anti-NOX2, anti- 8-OHdG, anti-i-NOS, anti-Nitrotyrosine, anti-BCL2 and anti-SMAC/DIABLO boiling in 0.1 M Citric Acid buffer. The primary antibody was applied in 1:10 ratio for 8-OHdG, in 1:50 ratio for NOX2, i-NOS, BCL2, in 1:100 ratio for Nitrotyrosine and SMAC/DIABLO, in 1:600 ratio for TNF-α, and incubated for 120 min at 20 °C. The detection system utilized was the LSAB + kit (Dako, Copenhagen, Denmark), a refined avidin-biotin technique in which a biotinylated secondary antibody reacts with several peroxidase-conjugated streptavidin molecules. For TUNEL assay (Apotag Plus Peroxidase *In Situ* Apoptosis Detection Kit, Chemicon (Millipore), Billerica, MA, USA) sections were pre-treated with Proteinase K (Sigma-Aldrich, Buchs, Switzerland) (20 μg/ml) for 15 min. at 20 °C; covered and incubated with the TdT enzyme; diluted in a ratio of 30% in reaction buffer for 60 min. at 38 °C; put in a coplin jar containing working strength stop/wash buffer; shaken for 15 seconds and incubated for 10 minutes at 20 °C, and covered and incubated with anti-digoxigenin conjugate for 30 minutes at 20 °C. The sections were counterstained with Mayer’s haematoxylin, dehydrated, cover slipped and observed in a Leica DM6000 optical microscope (Leica, Cambridge, UK). Apoptosis was measured by TUNEL assay. In order to determine the fraction of myocyte nuclei labelled by TUNEL, the number of myocyte nuclei per unit area of tissue was calculated by counting an average of 10 fields, 1.4 mm^2^ each, at a magnification of 10x in each area of myocardium sampled. The percentage of apoptotic myocyte nuclei was determined. For semiquantitative analysis, slides were scored in a blind manner by two observers (MN, ET). The intensity of immunopositive expression was assessed semiquantitatively on a scale of 0–4 as follows: 0 = no immunoreactivity, 1 = mild immunopositivity in scattered cells, 2 = immunopositivity in up to one third of cells, 3 = immunopositivity in up to half the cells and 4 = strong immunopositivity in the majority or all of the cells. In cases of divergent scoring, a third observer (VF) decided the final category.

### Statistical analysis

Results were expressed as mean ± SD. The comparison between groups was conducted using the comparison Student’s T test. A value of P < 0.05 was considered statistically significant.

## Additional Information

**How to cite this article**: Turillazzi, E. *et al*. Myocardial oxidative damage is induced by cardiac Fas-dependent and mitochondria-dependent apoptotic pathways, in human cocaine-related overdose. *Sci. Rep.* **7**, 44262; doi: 10.1038/srep44262 (2017).

**Publisher's note:** Springer Nature remains neutral with regard to jurisdictional claims in published maps and institutional affiliations.

## Figures and Tables

**Table 1:** Cocaine blood level at the time of death.

Case	Substance*	Sample or tissue									
		Brain	Lung	Spleen	Liver	Kidney	Blood	Vitreous umor	Gastric content	Bile	Urine
1	Cocaine				2090	1470	1720				5330
	Ecgonine										
	Benzoil-ecgonine				3990	4810	4130				8230
2	Cocaine						96.77	3.8			147
	Ecgonine						46.2				
	Benzoil-ecgonine						52.4				
3	Cocaine				371.9	50.38	47.56				241.42
	Ecgonine				19.41	6.18	25.36				153.77
	Benzoil-ecgonine				59.14	4.08	27.27				744.23
4	Cocaine	7.14			6.06		40.7			40.26	51.89
	Ecgonine										
	Benzoil-ecgonine	3.31			13.09		22.22			4.11	95.7
5	Cocaine	20.13	16.27		25.11	25.16	80.03	4.16		233.34	811.98
	Ecgonine										
	Benzoil-ecgonine	8.07	13.02		69.98	21.59	41.77	5.89		600.2	570.37
6	Cocaine				2.28	1.02	1		35	4.12	12.01
	Ecgonine				3.02	1.23	0.88		0.21	5.94	80.18
	Benzoil-ecgonine				5.15	7.98	9.18		498.11	91.89	1829.16
7	Cocaine	89.9	111.8	77.7	144.9	89.94	98.8				21
	Ecgonine	43.6	95.4	5.1	21.2	5.8	55.6				4.3
	Benzoil-ecgonine	21.7	41.4	7.8	143.8	9.4	79.1				5.1
8	Cocaine	77.3	118.1	77.7	120.3	110.8	100.2				9.2
	Ecgonine	26.8	75.6	4.2	28.2	7.4	48.88				3.6
	Benzoil-ecgonine	16.6	30.4	5.5	111.1	7.7	70.6				4.2
9	Cocaine	79.6	130.8	76.8	80.2	80.1	80.8				10.8
	Ecgonine	50.2	70.2	5.2	19.5	6.2	36.6				4.3
	Benzoil-ecgonine	19,4	34.6	6.4	100.6	7.2	50.2				4.8

^*^U.M. mcg/ml/g.

**Table 2:** Lipid peroxidation, ascorbic acid, and GSH/GSSG ratio results.

	Study group	Control group
MDA (nmol/g tissue)	11.27 ± 4.29*	4.22 ± 2.553
AA (nmol/g tissue)	12,93 ± 3,451*	29,51 ± 12,454
GSH/GSSG	7.6 ± 1.99**	60.0 ± 15.66

Values are presented as the mean ± SD of study group (n = 9) and control group cases (n = 9). ^*^ p < 0.05; **p < 0.01 study group vs control group.

**Table 3:** Statistical analysis of the immunohistochemical findings and gradation of the immunohistochemical reactions.

	Control group	Cocaine group	Statistical value Cocaine vs Control
Anti-iNOs	+/−	+++	***
Anti-NOX2	+/−	+++	***
Anti – Nitrotyrosine	+	+++	***
Anti-8OHdG	+/−	+++	***
Anti - Bcl-2	+/−	++	**
Smac/DIABLO	+/−	+++	***
Anti-TNFα	+	+++	***
TUNEL assay	+	+++	***

Responses about iNOs, NOX2, Nytrotirosine, 8OHdG, Bcl-2, and apoptosis with TUNEL assay in heart specimens. Results were expressed as mean ± SD. The comparison between groups was conducted using the comparison Student’s *t* test. A value of *p* < 0.05 was considered statistically significant. NS: *p* > 0.05; *p < 0.05; **p < 0.01; ***p < 0.001. Intensity of immunopositivity was assessed semiquantitatively in the scale 0–4 as follows: −: no immunoreactivity (0%); +: mild immunopositivity in scattered cells (10%); ++: immunopositivity in up to one third of cells (33%); +++: immunopositivity in up to two- third of cells (70%) and ++++: strong immunopositivity in the majority or all cells (100%). In cases of divergent scoring, a third observer decided the final category.
